# Supplementary material for: Structural and thermodynamic insights into antibody light chain tetramer formation through 3D domain swapping
Source: Nat Commun. 2023 Dec 8;14:7807. doi: 10.1038/s41467-023-43443-4 (PMC10709643; doi:10.1038/s41467-023-43443-4)
Supplement: Supplementary file 1 — Supplementary Information [file 41467_2023_43443_MOESM1_ESM.pdf]

Supplementary Information

Structural and Thermodynamic Insights into Antibody Light Chain Tetramer Formation through 3D Domain Swapping

Takahiro Sakai[1], Tsuyoshi Mashima[1], Naoya Kobayashi[1], Hideaki Ogata[2], Lian Duan[3,4], Ryo Fujiki[3], Kowit Hengphasatporn[3], Taizo Uda[5], Yasuteru Shigeta[3], Emi Hifumi[6] & Shun Hirota[1*]

[1] Division of Materials Science, Graduate School of Science and Technology, Nara Institute of Science and Technology

[2] Graduate School of Science, University of Hyogo

[3] Center for Computational Sciences, University of Tsukuba

[4] Graduate School of Pure and Applied Sciences, University of Tsukuba

[5] Nanotechnology Laboratory, Institute of Systems, Information Technologies and Nanotechnologies (ISIT)

[6] Institute for Research Management, Oita University

1. Supplementary Figures

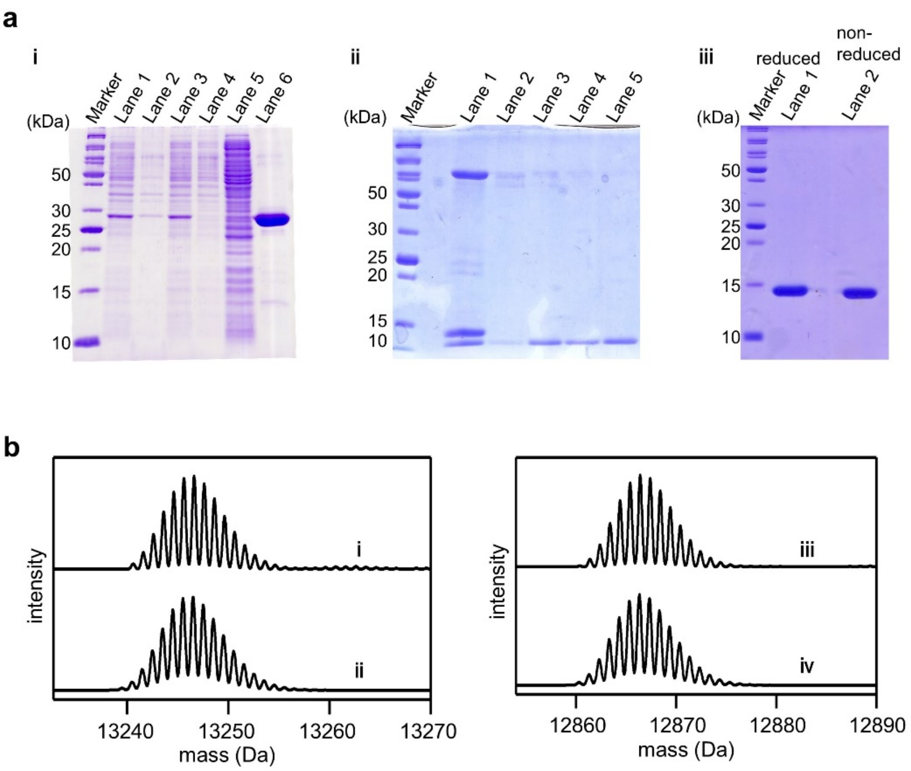

Supplementary Fig. 1 | SDS-PAGE and LC/ESI-QTOF-MS analyses. a SDS-PAGE gel image of: i) #4C214AinsCS^{TEV}, ii) TEV protease-cleaved #4C214AinsCS^{TEV}, iii) #4C$_L$. i) Lane 1: *E.coli* cell solution after sonication, Lane2: Pellet of sonicated *E.coli* cell solution, Lane 3: Supernatant of sonicated *E.coli* cell solution, Lane 4: Eluted solution after passing the sample through the Ni-NTA agarose column, Lane 5: Eluted solution after passing 50 mM Tris–HCl buffer, pH 8.0, through the Ni-NTA agarose column, Lane 6: Eluted solution after passing 50 mM Tris–HCl buffer, pH 8.0, containing 0.25 M NaCl and 0.3 M imidazole through the Ni-NTA agarose column. ii) Lane 1: Sample after the reaction of #4C214AinsCS^{TEV} with TEV protease, Lane 2: Eluted solution after passing the sample through the Ni-NTA agarose column, Lane 3: Eluted solution after passing the sample through the Ni-NTA agarose column twice, Lane 4: Eluted solution after passing the sample through the Ni-NTA agarose column three times, Lane 5: Eluted solution after passing 50 mM Tris–HCl buffer, pH 8.0, through the Ni-NTA agarose column, in which the proteins containing the His-tag were removed. iii) Lane 1: #4C$_L$ post-treatment with 2-mercaptoethanol, Lane 2: #4C$_L$ without 2-mercaptoethanol treatment. **b** LC/ESI-QTOF-MS spectra: i) #4V$_L$ (measured), ii) #4V$_L$ (calculated), iii) #4C$_L$ (measured), iv) #4C$_L$ (calculated).

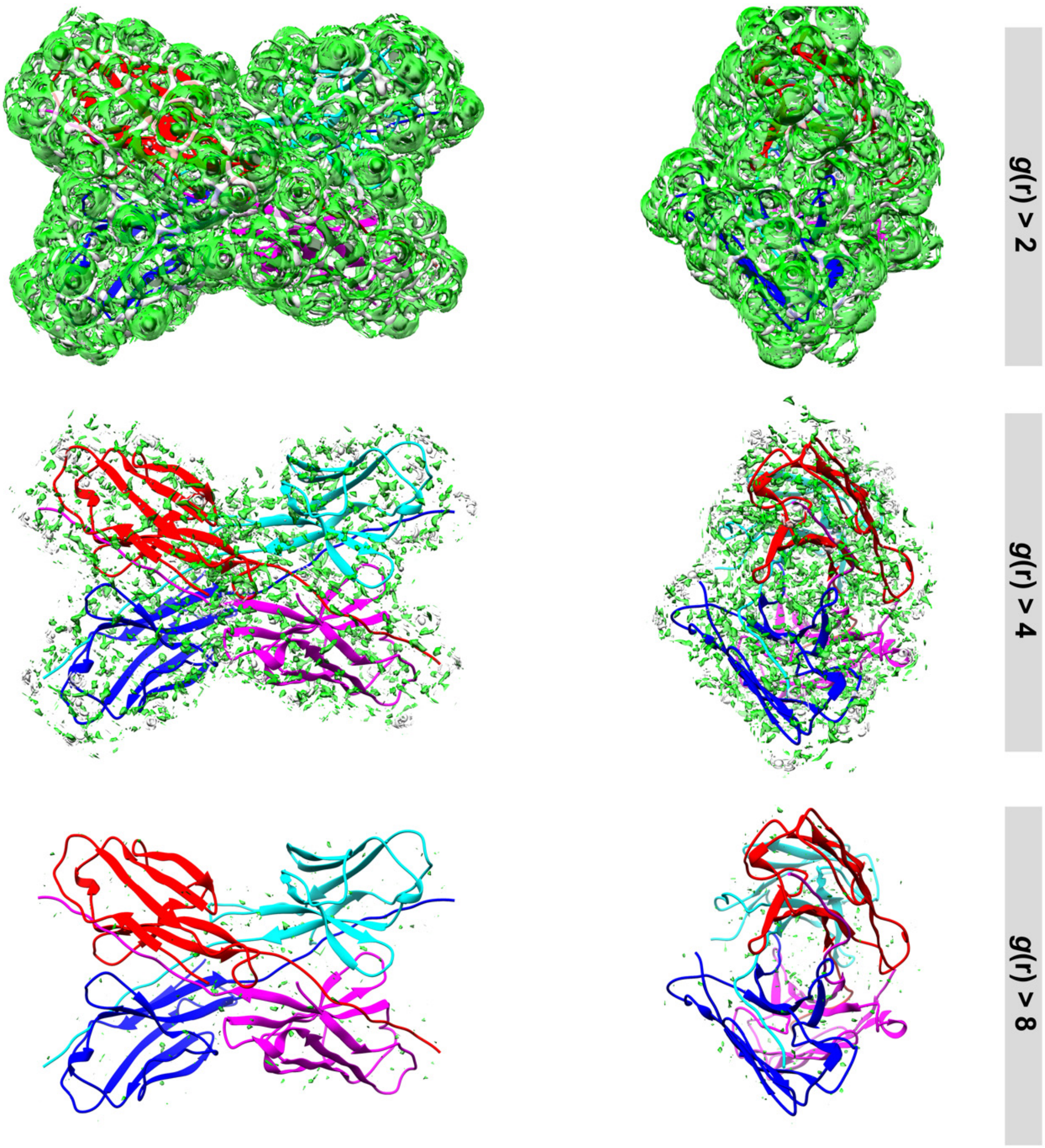

Supplementary Fig. 2 | 3D distribution of water molecules (g(r)) (oxygen and hydrogen atoms) around the #4V_L tetramer. The water molecules are illustrated in green with g(r) values greater than 2, 4, and 8.

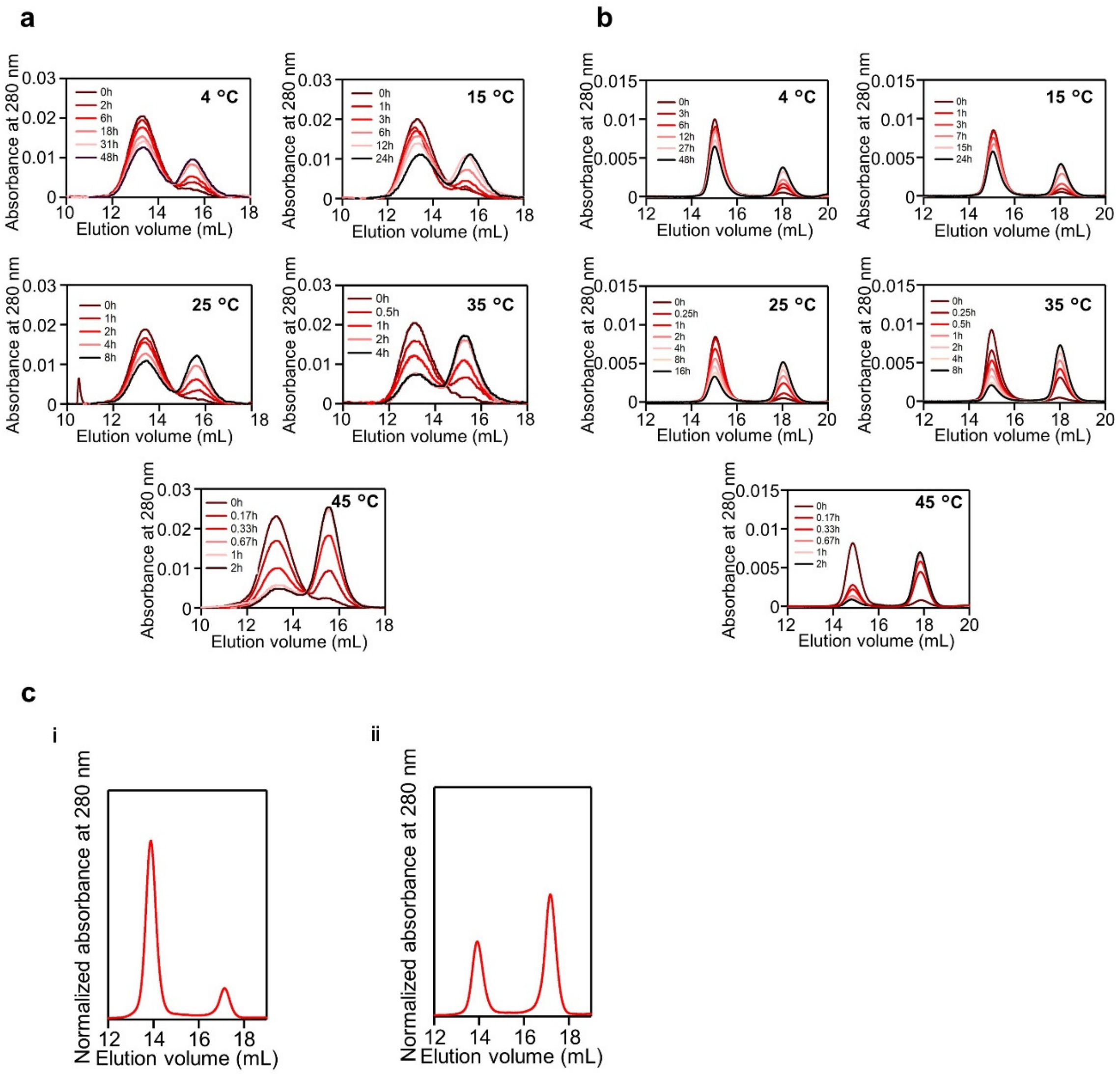

Supplementary Fig. 3 | SEC chromatograms. a Time-course tracking of elution profiles of #4C214A after dilution from 300 to 6 μM at 4–45 °C. **b** Time-course tracking of elution profiles of #4V$_L$ after dilution from 300 to 6 μM at 4–45 °C. **c** Elution profiles of #4V$_L$ at different concentrations: i) 100 μM, ii) 10 μM.

#4C214A

NcoI — CDR1 (24, 27 a b c d e f 28, 30) — 1, 10, 35

MDVVMTQTPLSLPVTPGEPASISCRSTQSLLDSDGVNPSFDWYLQK

40, 50 CDR2, 57, 60, 70, 80

PGQSPQLLIHRGFYRASGVPDRFSGSGSGTDFTLRISRVEAEDVGV

85, 89 CDR3, 98, 100, 108, 110, 120, 130

YYCMQRIEFPLTFGGGTKVEIKRTVAAPSVFIFPPSDEQLKSGTAS

132, 140, 150, 160, 170

VVCLLNNFYPREAKVQWKVDNALQSGNSQESVTEQDSKDSTYSLSS

178, 180, 190, 200, 210, 214 — XhoI — His tag

TLTLSKADYEKHKVYACEVTHQGLSSPVTKSFNRGEALEHHHHHH

Supplementary Fig. 4 | Amino acid sequence of #4C214A.

#4C214AinsCS^{TEV}

NcoI
CDR1
MDVVMTQTPLSLPVTPGEPASISCRSTQSLLDSDGVNPSFDWYLQK

CDR2
PGQSPQLLIHRGFYRASGVPDRFSGSGSGTDFTLRISRVEAEDVGV

CDR3
TEV cleavage site
YYCMQRIEFPLTFGGGTKVEIKENLYFQGRTVAAPSVFIFPPSDEQ

LKSGTASVVCLLNNFYPREAKVQWKVDNALQSGNSQESVTEQDSKD

XhoI
STYSLSSTLTLSKADYEKHKVYACEVTHQGLSSPVTKSFNRGEALE

His tag
HHHHHH

#4V$_L$

NcoI
CDR1
MDVVMTQTPLSLPVTPGEPASISCRSTQSLLDSDGVNPSFDWYLQK

CDR2
PGQSPQLLIHRGFYRASGVPDRFSGSGSGTDFTLRISRVEAEDVGV

CDR3
TEV cleavage site (N)
YYCMQRIEFPLTFGGGTKVEIKENLYFQ

#4C$_L$

TEV clevage site (C)
GRTVAAPSVFIFPPSDEQLKSGTASVVCLLNNFYPREAKVQWKVDNA

LQSGNSQESVTEQDSKDSTYSLSSTLTLSKADYEKHKVYACEVTHQG

XhoI
His tag
LSSPVTKSFNRGEALEHHHHHHH

Supplementary Fig. 5 | Amino acid sequence of #4C214AinsCS^{TEV}, #4V$_L$, and #4C$_L$.

FW primer

GCCCTGAAAGTACAGGTTCTCCTTGATCTCCACCTTGGTCC

RV primer

CGAACTGTGGCTGCACCATCTGTCTTCATCTTCCCGCCATC

Supplementary Fig. 6 | Primer sequences of #4C214AinsCS^{TEV}.

TEV protease

Start codon MBP

MKIEEGKLVIWINGDKGYNGLAEVGKKFEKDTGIKVTVEHPDKLEEK

FPQVAATGDGPDIIFWAHDRFGGYAQSGLLAEITPDKAFQDKLYPFT

WDAVRYNGKLIAYPIAVEALSLIYNKDLLPNPPKTWEEIPALDKELK

AKGKSALMFNLQEPYFTWPLIAADGGYAFKYENGKYDIKDVGVDNAG

AKAGLTFLVDLIKNKHMNADTDYSIAEAAFNKGETAMTINGPWAWSN

IDTSKVNYGVTVLPTFKGQPSKPFVGVLSAGINAASPNKELAKEFLE

NYLLTDEGLEAVNKDKPLGAVALKSYEEELAKDPRIAATMENAQKGE

IMPNIPQMSAFWYAVRTAVINAASGRQTVDEALKDAQTNSSSNNNNN

Linker TEV protease

NNNNNLGIEGRGSSGESLFKGPRDYNPISSTICHLTNESDGHTTSLY

GIGFGPFIITNKHLFRRNNGTLLVQSLHGVFKVKNTTTLQQHLIDGR

DMIIIRMPKDFPPFPQKLKFREPQREERICLVTTNFQTKSMSSMVSD

TSCTFPSSDGIFWKHWIQTKDGQCGSPLVSTRDGFIVGIHSASNFTN

TNNYFTSVPKNFMELLTNQEAQQWVSGWRLNADSVLWGGHKVFMVKP

XhoI His tag

EEPFQPVKEATQLMNGSLEHHHHHH

Supplementary Fig. 7 | Amino acid sequence of TEV protease.

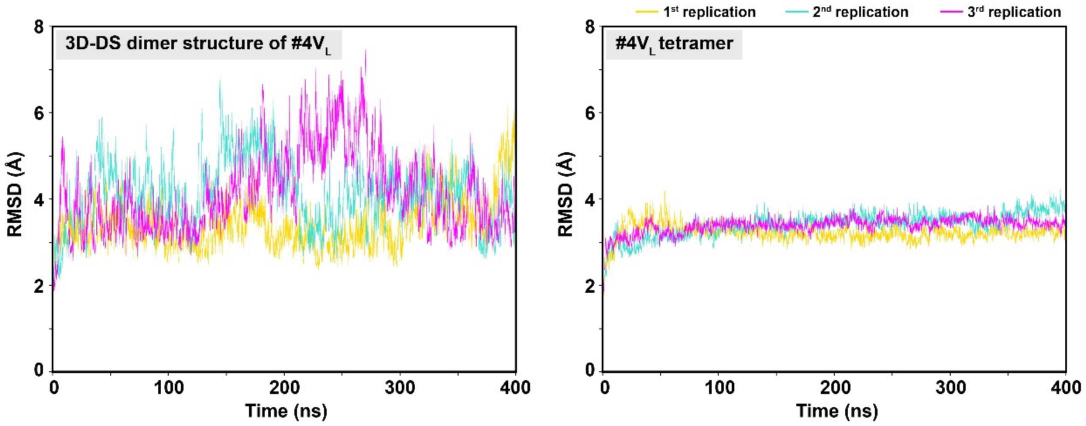

Supplementary Fig. 8 | Root mean square deviation (RMSD) analysis. RMSD was calculated on the Cα carbon atom coordinates over the 400 ns-MD trajectories obtained from three replicates of the 3D-DS dimer and tetramer of #4VL.

2. Supplementary Tables

Supplementary Table 1 | Tetramer ratio and dissociation constants of #4C214A and #4V_L at protein concentrations ranging from 3–300 µM.[a]

Concentration (µM)	#4C214A		#4V_L	
	Tetramer ratio (%)	K_D (M^3)	Tetramer ratio (%)	K_D (M^3)
3	19	2.4×10^{-16}	-	-
10	55	3.0×10^{-16}	30	3.1×10^{-15}
30	79	2.7×10^{-16}	64	2.8×10^{-15}
100	91	2.9×10^{-16}	84	3.0×10^{-15}
300	96	2.9×10^{-16}	93	3.0×10^{-15}

[a] at 277 K.

Supplementary Table 2 | Dissociation constants of #4C214A tetramer to monomers at temperatures ranging from 4–45 °C.

Temperature (°C)	K_D (M^3)	
	#4C214A	#4V_L
4	3.4×10^{-17}	4.4×10^{-17}
15	5.8×10^{-17}	6.4×10^{-17}
25	1.3×10^{-16}	1.9×10^{-16}
35	4.5×10^{-16}	8.1×10^{-16}
45	2.3×10^{-15}	3.9×10^{-15}

Supplementary Table 3 | Statistics of data collection and structure refinement of #4V$_L$ (PDB: 8KAD).

Data collection	
X-ray source	SPring-8 (BL45XU)
Wavelength (Å)	1.0000
Space group	$P6_522$
Unit cell parameters	
a, b, c (Å)	52.78, 52.78, 260.29
α, β, γ (º)	90.00, 90.00, 120.00
Resolution (Å)	45.71 – 2.00 (2.05 – 2.00)
Number of total reflections	138634 (10485)
Number of unique reflections	15551 (1103)
Multiplicity	8.9 (9.5)
Completeness (%)	99.9 (100.0)
$\langle I/\sigma(I) \rangle$	2.77 (1.4)
$CC_{1/2}$	95.2 (56.3)
R_{meas} (%)	34.6 (107.1)

Refinement	
Resolution (Å)	45.71 – 2.00
Number of reflections (working set)	14773
Number of reflections (test set)	778
R_{work}[a]	0.253
R_{free}[a]	0.296
Number of atoms in an asymmetric unit	
Protein	1696
Solvent	88
Average B factors (Å^2)	
Protein	46.3
Solvent	47.2
Ramachandran plot (%)	
Favored	97.65
Allowed	1.88
Outlier	0.47
RMSD, bonds (Å)	0.008
RMSD, angles (°)	1.16

Statistics for the highest-resolution shell are given in parentheses.

[a] $R_{\mathrm{work}} = \Sigma_{\mathrm{hkl}} \,|\,|F_{\mathrm{obs}}| - k|F_{\mathrm{calc}}|\,|\,(\Sigma_{\mathrm{hkl}} |F_{\mathrm{obs}}|)^{-1}$, k: scaling factor. R_{free} was computed identically, except where all reflections belong to a test set of 5 % of randomly selected data.

Supplementary Table 4 | Software and Codes used in this work.

Function	Software/Code	Source
Protein preparation	tLeap and SANDER	https://ambermd.org/
MD simulations	pmemd.cuda	https://ambermd.org/
Hydrogen bond, water shell, and RMSD analyses	Cpptraj	https://ambermd.org/
Interaction analysis	PyContact	https://pycontact.github.io/
	RING	https://ring.biocomputingup.it
Visualization	VMD version 1.9.3	https://www.ks.uiuc.edu/Research/vmd/
	UCSF Chimera 1.16	https://www.cgl.ucsf.edu/chimera/
3D-RISM	RISMiCal software	https://kyushu-u.elsevierpure.com/ja/publications/the-reference-interaction-site-model-integrated-calculator-rismic
