# Supplementary material for: Structural and thermodynamic insights into antibody light chain tetramer formation through 3D domain swapping
Source: Nat Commun. 2023 Dec 8;14:7807. doi: 10.1038/s41467-023-43443-4 (PMC10709643; doi:10.1038/s41467-023-43443-4)
Supplement: Supplementary file 3 — Reporting Summary [file 41467_2023_43443_MOESM3_ESM.pdf]

nature portfolio

Corresponding author(s): Shun Hirota

Last updated by author(s): Oct 25, 2023

Reporting Summary

Nature Portfolio wishes to improve the reproducibility of the work that we publish. This form provides structure for consistency and transparency in reporting. For further information on Nature Portfolio policies, see our Editorial Policies and the Editorial Policy Checklist.

Statistics

For all statistical analyses, confirm that the following items are present in the figure legend, table legend, main text, or Methods section.

n/a	Confirmed	
☐	☒	The exact sample size (n) for each experimental group/condition, given as a discrete number and unit of measurement
☐	☒	A statement on whether measurements were taken from distinct samples or whether the same sample was measured repeatedly
☒	☐	The statistical test(s) used AND whether they are one- or two-sided<br>*Only common tests should be described solely by name; describe more complex techniques in the Methods section.*
☒	☐	A description of all covariates tested
☒	☐	A description of any assumptions or corrections, such as tests of normality and adjustment for multiple comparisons
☐	☒	A full description of the statistical parameters including central tendency (e.g. means) or other basic estimates (e.g. regression coefficient) AND variation (e.g. standard deviation) or associated estimates of uncertainty (e.g. confidence intervals)
☒	☐	For null hypothesis testing, the test statistic (e.g. F, t, r) with confidence intervals, effect sizes, degrees of freedom and P value noted<br>*Give P values as exact values whenever suitable.*
☒	☐	For Bayesian analysis, information on the choice of priors and Markov chain Monte Carlo settings
☒	☐	For hierarchical and complex designs, identification of the appropriate level for tests and full reporting of outcomes
☒	☐	Estimates of effect sizes (e.g. Cohen's d, Pearson's r), indicating how they were calculated

Our web collection on statistics for biologists contains articles on many of the points above.

Software and code

Policy information about availability of computer code

Data collection	X-ray diffraction data for #4VL were obtained at the BL45XU beamline at SPring-8, Japan. Data collection was performed automatically via the ZOO system (Acta. Crystallogr. D Struct. Biol., 75, 138-150 (2019)).
Data analysis	For structural data processing, we used the following programs: KAMO system and the XDS program package (version Feb 5, 2021). Molecular replacement was carried out in Phaser (included in the PHENIX program). Refinement was carried out in PHENIX 1.13_2998 and model building was carried out in COOT (version 0.9.6). The atomic coordinates of the #4VL structure was predicted by AlphaFold2 (Nature 596, 583-589 (2021)).<br>For MD simulation, the atomic coordinates of the #4VL tetramer that have been deposited to the PDB under accession code 8KAD was utilized for structural preparation using tLeap within Ambertools 21. The pmemd.cuda module within Amber 20 was employed for MD simulations. MD trajectories (hydrogen bond and water shell analyses) were analyzed through the cpptraj program within the Amber 20 package. The PyContact (https://pycontact.github.io/) and RING (https://ring.biocomputingup.it/) software tools were used to assess hydrogen bond and hydrophobic interactions. Visualization of 3D structures was achieved using VMD (version 1.9.3) and UCSF Chimera 1.6. Solvation-free energy calculations were performed utilizing the RISMiCal software package for 3D-RISM analysis, which can be accessed at https://kyushu-u.elsevierpure.com/ja/publications/the-reference-interaction-site-model-integrated-calculator-rismic.<br>The thermodyanamic data were fitted using Igor Pro (version 6.01).<br>The figures of the structural models were prepared by PYMOL (version 2.4.0).

For manuscripts utilizing custom algorithms or software that are central to the research but not yet described in published literature, software must be made available to editors and reviewers. We strongly encourage code deposition in a community repository (e.g. GitHub). See the Nature Portfolio guidelines for submitting code & software for further information.

Data

Policy information about availability of data

All manuscripts must include a data availability statement. This statement should provide the following information, where applicable:

- Accession codes, unique identifiers, or web links for publicly available datasets
- A description of any restrictions on data availability
- For clinical datasets or third party data, please ensure that the statement adheres to our policy

The data that support this study are available from the corresponding author upon request. The atomic coordinates of the #4VL tetramer have been deposited to the Protein Data Bank under accession code 8KAD [http://doi.org/10.2210/pdb8KAD/pdb]. Plasmids are available from the corresponding author on a reasonable request.

Research involving human participants, their data, or biological material

Policy information about studies with human participants or human data. See also policy information about sex, gender (identity/presentation), and sexual orientation and race, ethnicity and racism.

Reporting on sex and gender	N/A
Reporting on race, ethnicity, or other socially relevant groupings	N/A
Population characteristics	N/A
Recruitment	N/A
Ethics oversight	N/A

Note that full information on the approval of the study protocol must also be provided in the manuscript.

Field-specific reporting

Please select the one below that is the best fit for your research. If you are not sure, read the appropriate sections before making your selection.

[x] Life sciences [ ] Behavioural & social sciences [ ] Ecological, evolutionary & environmental sciences

For a reference copy of the document with all sections, see nature.com/documents/nr-reporting-summary-flat.pdf

Life sciences study design

All studies must disclose on these points even when the disclosure is negative.

Sample size	The sample size was determined in preliminary experiments.
Data exclusions	No data was excluded from the analysis.
Replication	Each analysis was performed at least 3 times. #4 antibody light chain was already used and validated in several references (FASEB J., 30, 895-908 (2016); FASEB J., 31, 1668-1677 (2017); Antibody Engineering (Chapter 10, 2018, Edited by Thomas Boldicke, InTech publishers, London, UK).
Randomization	Randomization is not relevant to the study.
Blinding	Blinding is not relevant to the study.

Reporting for specific materials, systems and methods

We require information from authors about some types of materials, experimental systems and methods used in many studies. Here, indicate whether each material, system or method listed is relevant to your study. If you are not sure if a list item applies to your research, read the appropriate section before selecting a response.

Materials & experimental systems

n/a	Involved in the study	
[ ]	[x]	Antibodies
[x]	[ ]	Eukaryotic cell lines
[x]	[ ]	Palaeontology and archaeology
[x]	[ ]	Animals and other organisms
[x]	[ ]	Clinical data
[x]	[ ]	Dual use research of concern
[x]	[ ]	Plants

Methods

n/a	Involved in the study	
[x]	[ ]	ChIP-seq
[x]	[ ]	Flow cytometry
[x]	[ ]	MRI-based neuroimaging

Antibodies

Antibodies used	DNAs encoding human antibody light chains was amplified and cloned from germline gene of subgroup II. The DNA fragment of an antibody light chain #4 was derived from germline gene O11/O1.
Validation	#4 antibody light chain was already used and validated in several references (FASEB J., 30, 895-908 (2016); FASEB J., 31, 1668-1677 (2017); Antibody Engineering (Chapter 10, 2018, Edited by Thomas Boldicke, InTech publishers, London, UK).
